# Glucocorticoid Receptor 1B and 1C mRNA Transcript Alterations in Schizophrenia and Bipolar Disorder, and Their Possible Regulation by GR Gene Variants

**DOI:** 10.1371/journal.pone.0031720

**Published:** 2012-03-13

**Authors:** Duncan Sinclair, Janice M. Fullerton, Maree J. Webster, Cynthia Shannon Weickert

**Affiliations:** 1 Schizophrenia Research Institute, Sydney, New South Wales, Australia; 2 Neuroscience Research Australia, Sydney, New South Wales, Australia; 3 School of Psychiatry, University of New South Wales, Sydney, New South Wales, Australia; 4 School of Medical Sciences, University of New South Wales, Sydney, New South Wales, Australia; 5 Stanley Laboratory of Brain Research, Uniformed Services University of the Health Sciences, Bethesda, Maryland, United States of America; Chiba University Center for Forensic Mental Health, Japan

## Abstract

Abnormal patterns of HPA axis activation, under basal conditions and in response to stress, are found in individuals with schizophrenia and bipolar disorder. Altered glucocorticoid receptor (GR) mRNA and protein expression in the dorsolateral prefrontal cortex (DLPFC) in psychiatric illness have also been reported, but the cause of these abnormalities is not known. We quantified expression of GR mRNA transcript variants which employ different 5′ promoters, in 35 schizophrenia cases, 31 bipolar disorder cases and 34 controls. We also explored whether sequence variation within the NR3C1 (GR) gene is related to GR mRNA variant expression. Total GR mRNA was decreased in the DLPFC in schizophrenia cases relative to controls (15.1%, *p*<0.0005) and also relative to bipolar disorder cases (8.9%, *p*<0.05). GR-1B mRNA was decreased in schizophrenia cases relative to controls (20.2%, *p*<0.05), while GR-1C mRNA was decreased in both schizophrenia and bipolar disorder cases relative to controls (16.1% and 17.2% respectively, both *p*<0.005). A dose-dependent effect of rs10052957 genotype on GR-1B mRNA expression was observed, where CC homozygotes displayed 18.4% lower expression than TC heterozygotes (*p*<0.05), and 31.8% lower expression than TT homozygotes (*p*<0.005). Similarly, a relationship between rs6190 (R23K) genotype and GR-1C expression was seen, with 24.8% lower expression in GG homozygotes than GA heterozygotes (*p*<0.01). We also observed an effect of rs41423247 (Bcl1) SNP on expression of 67 kDa GRα isoform, the most abundant GRα isoform in the DLPFC. These findings suggest possible roles for the GR-1B and GR-1C promoter regions in mediating GR gene expression changes in psychotic illness, and highlight the potential importance of sequence variation within the NR3C1 gene in modulating GR mRNA expression in the DLPFC.

## Introduction

Appropriate responses to stress, mediated by the hypothalamic-pituitary-adrenal (HPA) axis, are fundamental to survival and adaptation [1]. Conversely, inappropriate and prolonged stress responses have been implicated in the onset and symptoms of psychiatric illnesses including schizophrenia and bipolar disorder. Among the possible mediators of the maladaptive stress response is the glucocorticoid receptor (GR), which binds the HPA axis stress hormone cortisol.

Multiple studies have identified abnormalities of GR in the brains of individuals with schizophrenia and bipolar disorder. Decreased expression of total GR mRNA is evident in the dorsolateral prefrontal cortex (DLPFC) in schizophrenia [2], [3], in the temporal cortex, hippocampus and amygdala in schizophrenia and bipolar disorder [2], [4], and in the entorhinal cortex in bipolar disorder [2]. At the protein level, increased abundance of a truncated GRα isoform, putative GRα-D1, in the DLPFC in schizophrenia and bipolar disorder has also been recently reported by our group [3]. These abnormalities of GR expression occur in the context of broader HPA axis dysregulation, which is observed in many individuals with schizophrenia and bipolar disorder and is characterised by increased cortisol secretion and diminished dexamethasone suppression in the illnesses [5], [6].

One of the possible mechanisms driving HPA axis dysfunction and GR disturbances in psychiatric illness is genetic variation in the GR (NR3C1) gene. GR gene polymorphisms have been shown to impact HPA axis stress responsiveness [7]–[10], and may contribute to an individual's vulnerability to maladaptive stress responses and to their propensity to develop psychiatric illness [11]. This hypothesis is supported by evidence of association of genetic polymorphisms in the GR gene with risk for bipolar disorder [12] and major depression [13], [14]. Other stress signalling genes such as FKBP5 have also been implicated in bipolar disorder [15], [16]. In the GR gene, polymorphisms have been identified within the coding, intronic and untranslated 5′ and 3′ regions. The mechanisms through which these polymorphisms may mediate their effects on vulnerability to psychiatric illness have not been established. We hypothesized that GR genotype may be linked to changes in GR mRNA levels in the human brain, and that this may contribute to risk of developing psychiatric illness.

**Figure 1:**
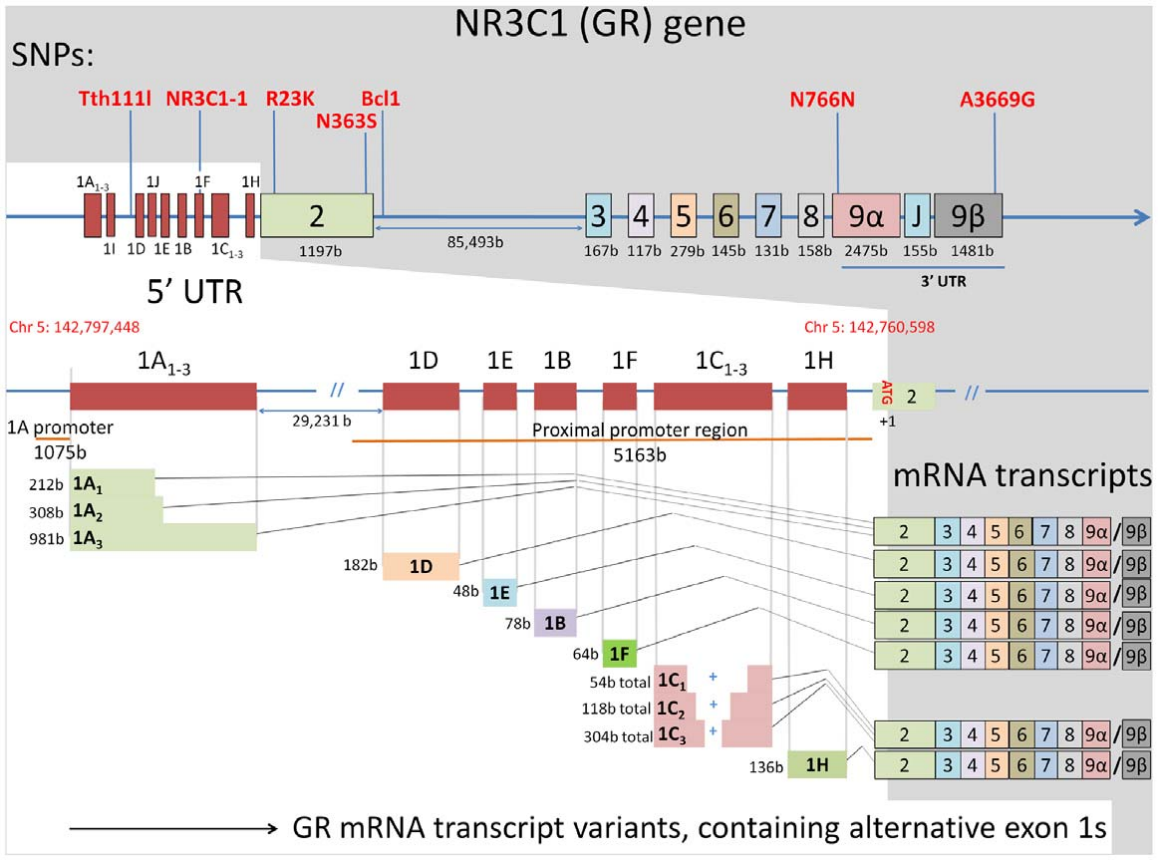
Structure of the GR gene (NR3C1). Map illustrates functional SNPs present in the gene and the generation of GR mRNA variants by inclusion of various exon 1 s in the mRNA 5′ UTR. SNP- single nucleotide polymorphism, UTR- untranslated region, b- base pairs, Chr- chromosome.

Since multiple different promoters may be used to synthesise alternative GR mRNA transcripts variants, examination of these variants may provide clues about the mechanisms underlying GR mRNA dysregulation in schizophrenia. Thus far, investigations of GR mRNA expression in psychiatric illness have focused on total GR mRNA abundance, employing Taqman qPCR probes or riboprobes which do not distinguish between GR mRNA transcript variants. As a result, little is known about the specific mRNA transcripts which are altered in psychiatric illness. Upstream of the GR gene (NR3C1) lie promoter regions which regulate tissue-specific expression of the thirteen GR exon 1 mRNA variants identified to date [17]–[21] (Figure 1). Numerous GR exon 1 mRNA variants are expressed in the human hippocampus [17], but it is not known which variants are expressed in other brain regions including the prefrontal cortex.

To date, while GR mRNA deficits have been identified in the DLPFC in schizophrenia, the evidence for such deficits in the DLPFC in bipolar disorder is inconclusive [2]. More generally, some GR abnormalities and aspects of HPA axis dysfunction are common to both schizophrenia and bipolar disorder, while other abnormalities are distinct to each illness [2], [4]–[6]. Examination of GR mRNA transcripts variant expression may provide a targeted means of identifying GR mRNA abnormalities in bipolar disorder, thus contributing to an understanding of common and distinct aspects of GR dysregulation in schizophrenia and bipolar disorder.

In this study, we explored GR splice variant mRNA expression in the DLPFC in both schizophrenia and bipolar disorder, using a cohort of 100 post-mortem samples. We sought to: 1) determine which GR alternative 5′ exons are employed in the adult human DLPFC, 2) determine whether expression levels of these alternative GR mRNA transcript variants are altered in schizophrenia and/or bipolar disorder cases relative to controls; and 3) explore whether NR3C1 polymorphisms may be associated with GR mRNA transcript and GRα protein isoform expression levels in human brain.

## Results

### Endpoint PCR detection of GR exon 1 transcript variants

**Figure 2:**
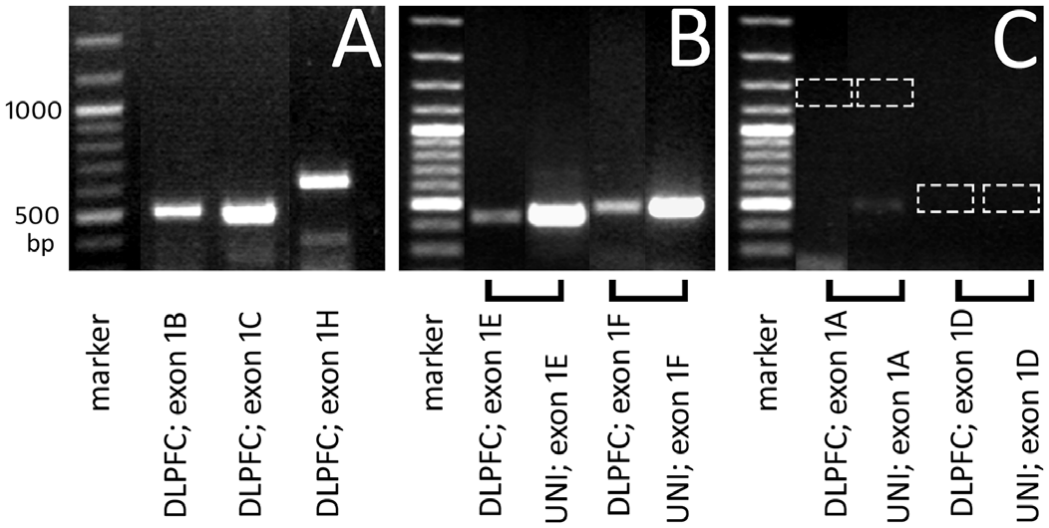
Endpoint PCR detection of GR exon 1 mRNA transcript variants in the DLPFC. A) strong amplification of GR-1B, GR-1C and GR-1H variants was evident in DLPFC cDNA, B) weaker amplification of GR-1E and GR-1F variants was observed in the DLPFC, but strong amplification was observed in universal human cDNA, C) GR-1A and GR-1D variants were detected neither in the DLPFC nor in universal cDNA. UNI- universal human cDNA.

Initially, to determine which specific GR exon 1 mRNA variants were expressed in the frontal cortex, endpoint PCR was performed with primers targeting GR transcripts containing exons 1A, 1B, 1C, 1D, 1E, 1F and 1H. The transcript variants GR-1B, GR-1C and GR-1H were found to be strongly expressed in pooled DLPFC cDNA (from schizophrenia, bipolar disorder and control cases) (Figure 2A). GR-1E and GR-1F were weakly expressed in pooled DLPFC cDNA, but were abundant in universal cDNA isolated from a panel of human tissues (Figure 2B). GR-1A and GR-1D could not be detected in DLPFC or universal cDNA (Figure 2C). Primers/probes targeting GR-1B, GR-1C, GR-1D, GR-1E, GR-1F and GR-1H were tested for use in qPCR analysis. Reliable amplification of the GR-1D, GR-1E and GR-1F transcripts was not achieved by qPCR, so we report quantitative analysis of pan GR, GR-1B, GR-1C and GR-1H mRNA expression.

### GR mRNA expression in the DLPFC in schizophrenia and bipolar disorder

Next, we determined whether DLPFC expression levels of pan GR, GR-1B, GR-1C and GR-1H mRNA transcripts were influenced by cohort demographic variables in the 100 cases analysed (Table 1). Pan GR and GR-1H mRNA expression did not significantly correlate with age, RIN, brain pH, PMI or brain weight. There were no significant correlations of GR-1-B and GR-1C mRNA expression with age, RIN or PMI, but there were significant positive correlations of GR-1B mRNA expression with brain weight (r = 0.25, *p*<0.05) and of GR-1C mRNA expression with brain pH (r = 0.22, *p*<0.05).

**Table 1:** Demographic details of cases from the Stanley Array cohort used to quantify GR exon 1 mRNA transcript variant expression.

	Control group	Bipolar disorder group	Schizophrenia group
number of cases	34	31	35
age (years)	43.8 (31–60)	44.9 (19–64)	42.6 (19–59)
gender	9F, 25M	16F, 15M	9F, 26M
hemisphere	16L, 18R	17L, 14R	17L, 18R
pH	6.61+/−0.27	6.46+/−0.28	6.47+/−0.24
PMI (hrs)	29.5+/−13.0	36.6+/−18.1	31.4+/−15.5
RIN	8.30+/−0.69	8.32+/−0.84	8.47+/−0.56
manner of death	natural = 34	natural = 17, suicide = 14	natural = 28, suicide = 7
age of onset (years)	-	24.8+/−9.0	21.3+/−6.1
duration of illness (years)	-	20.2+/−9.9	21.3+/−10.1
lifetime antipsychotics (fluphenazine equivalents, mg)	-	10297+/−23865	85004+/−100335
antidepressant use	yes = 0, no = 34	yes = 18, no = 13	yes = 9, no = 26
smoking status (around time of death)	yes = 9, no = 9, unknown = 16	yes = 14, no = 6, unknown = 11	yes = 23, no = 4, unknown = 8

Abbreviations: M- male, F- female, L- left, R- right, PMI- post-mortem interval, RIN- RNA integrity number. Data quoted are mean (range) +/− standard deviation.

**Figure 3:**
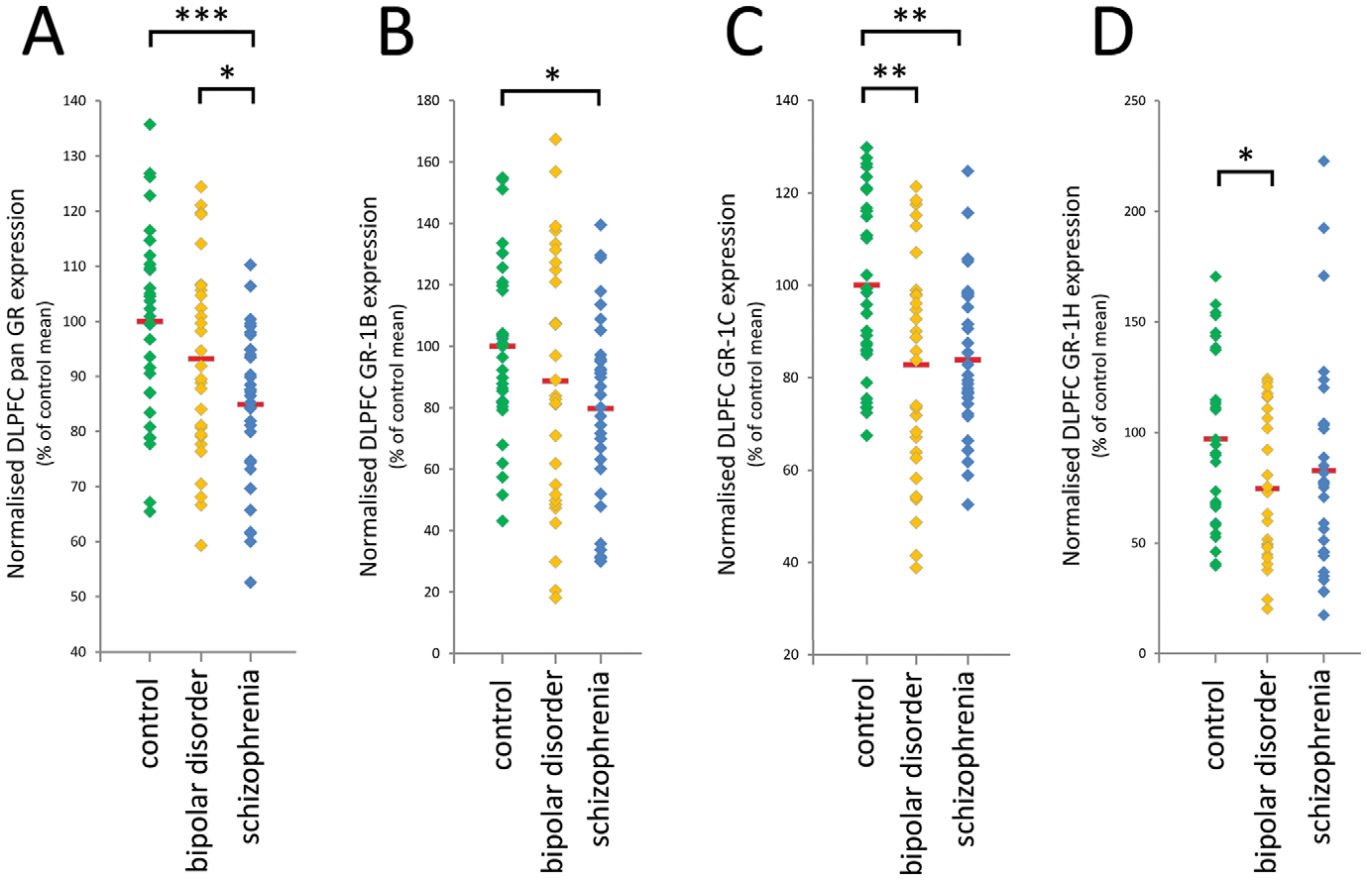
Expression of pan GR, GR-1B, GR-1C and GR-1H mRNA transcripts in the DLPFC. Control, bipolar disorder and schizophrenia cases from the Stanley Array cohort were used. A) Pan GR mRNA expression was 15.1% lower in schizophrenia cases relative to controls (*p*<0.0005), and 8.9% lower in schizophrenia cases relative to bipolar disorder cases (*p*<0.05). B) GR-1B mRNA expression also differed significantly between diagnostic groups, with 20.2% lower GR-1B expression in schizophrenia cases than controls (*p*<0.05). C) GR-1C mRNA expression was significantly decreased in schizophrenia (16.1%) and bipolar disorder (17.2%) cases relative to controls (both *p*<0.005). D) A significant effect of diagnosis GR-1H expression was not observed, but a planned post-hoc test revealed a significant difference between bipolar disorder and control groups (*p*<0.05). Green, orange and blue diamonds represent control, bipolar disorder and schizophrenia cases respectively. Red bars represent group means. * *p*<0.05, ** *p*<0.005, *** *p*<0.0005.

Analysis of pan GR mRNA revealed significant group differences in expression according to diagnosis (ANOVA F(2,92) = 6.96, *p*<0.005). Pan GR mRNA expression in the DLPFC was 15.1% lower in schizophrenia cases relative to controls by LSD post-hoc test ( *p*<0.0005), and 8.9% lower in schizophrenia cases relative to bipolar disorder cases (*p*<0.05; Figure 3). The 6.8% decrease in bipolar disorder cases relative to controls did not reach significance (*p* = 0.11).

GR-1B and GR-1C mRNA transcript expression also differed significantly between diagnostic groups. There was a significant effect of diagnosis on GR-1B mRNA expression [ANCOVA F(2, 92) = 3.16, *p*<0.05 (co-varying for brain weight); Figure 3B]. GR-1B expression was significantly decreased (20.2%) in schizophrenia cases relative to controls (*p*<0.05), and non-significantly decreased (11.3%) in bipolar disorder cases relative to controls (*p* = 0.18). A significant effect of diagnosis on GR-1C mRNA transcript expression was also observed [ANCOVA F(2, 91) = 5.59, *p* = 0.005 (co-varying for brain pH); Figure 3C]. GR-1C mRNA expression was significantly decreased in both schizophrenia (16.1%) and bipolar disorder (17.2%) cases relative to controls (both *p*<0.005). For GR-1H, there was a trend towards a diagnosis effect on GR-1H mRNA expression (ANOVA F(2, 88) = 2.26, *p* = 0.11; Figure 3D). In a planned comparison between bipolar disorder cases and controls, GR-1H mRNA expression was 22.6% lower in bipolar disorder cases than controls (*p*<0.05). There was no significant difference in GR-1H mRNA between schizophrenia cases and controls. Three schizophrenia cases were observed to have substantially higher GR-1H mRNA expression than the other schizophrenia cases in the cohort (Figure 3D), potentially impacting our ability to identify group differences.

Expression levels of pan GR mRNA were highly significantly correlated with levels of all GR exon 1 mRNA transcript variants measured (GR-1B, r = 0.69, *p*<1×10^−12^; GR-1C, r = 0.55, *p*<1×10^−7^; GR-1H, r = 0.36, *p*<1×10^−3^), suggesting that each contributed significantly to overall pan GR mRNA levels.

### Effects of suicide and other cohort demographic variables on GR mRNA expression

**Figure 4:**
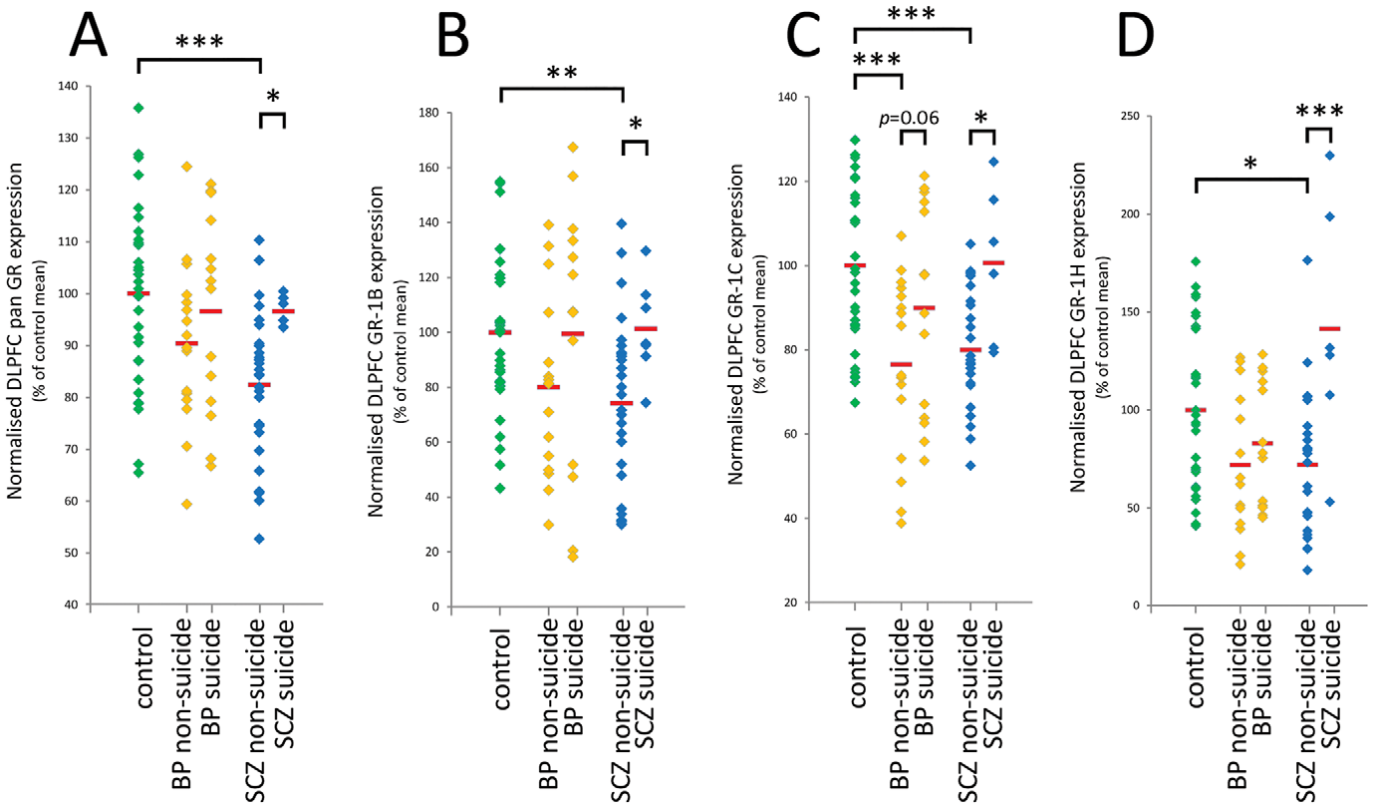
Effect of suicide on expression of GR mRNA transcripts in the DLPFC. Suicide-positive and suicide-negative control cases, bipolar disorder cases and schizophrenia cases were compared. A) Pan GR mRNA expression; B) GR-1B mRNA expression; C) GR-1C mRNA expression; D) GR-1H mRNA expression. For all transcripts, GR transcript expression was decreased in suicide-negative schizophrenia cases relative to suicide-positive schizophrenia cases and relative to controls (all suicide-negative). For the GR-1C mRNA transcript, expression was decreased in bipolar disorder as well as schizophrenia in suicide-negative cases relative to both suicide-positive cases and controls. Green, orange and blue diamonds represent control, bipolar disorder and schizophrenia cases respectively. Red bars represent group means. Abbreviations: BP- bipolar disorder, SCZ- schizophrenia. * *p*≤0.05, ** *p*<0.005, *** *p*<0.0005.

The relationship between manner of death and DLPFC GR mRNA transcript expression was investigated. When schizophrenia and bipolar disorder groups were subdivided according to suicide status, significant group differences in pan GR mRNA expression were observed (ANOVA (F(4, 90) = 4.83, *p*<0.005). Individuals with schizophrenia who had not died by suicide (suicide-negative) displayed 14.7% lower pan GR mRNA levels than individuals with schizophrenia who had died by suicide (suicide-positive) (*p* = 0.05) and 17.7% lower pan GR mRNA levels than controls (all suicide-negative) (*p*<0.0001) (Figure 4A). There was no difference in pan GR mRNA expression between suicide-positive schizophrenia cases and controls. In bipolar disorder, no differences between suicide-positive and suicide-negative cases were observed.

A similar relationship between suicide and expression of all individual GR mRNA transcript variants in schizophrenia cases was observed. Expression of GR-1B mRNA (ANCOVA (F(4, 90) = 2.79, *p*<0.05), GR-1C (ANCOVA (F(4, 89) = 5.11, *p*<0.001) and GR-1H mRNA (ANOVA (F(4, 87) = 5.34, p<0.005) varied significantly according to suicide status within diagnostic groups. GR-1B expression of suicide-negative schizophrenia cases was 26.8% lower than suicide-positive schizophrenia cases (*p*<0.05), and 25.8% lower than controls (*p*<0.005; Figure 4B). Similarly, GR-1C expression of suicide-negative schizophrenia cases was 20.6% lower than suicide-positive schizophrenia cases (*p*<0.05), and 20.0% lower than controls (*p*<0.0005; Figure 4C). In bipolar disorder, GR-1C mRNA expression was also impacted by suicide status, with 14.9% lower GR-1C mRNA levels in suicide-negative bipolar disorder cases than suicide-positive cases (trend *p* = 0.06), and 23.5% lower than controls (*p*<0.0005). Finally, GR-1H expression of suicide-negative schizophrenia cases was 48.8% lower than suicide-positive schizophrenia cases (*p*<0.0005), and 27.5% lower than controls (*p*<0.05; Figure 4D).

The effects of other cohort demographic variables (Table 1) on GR mRNA transcript variant expression were also explored. No significance differences in pan GR, GR-1B, GR-1C or GR-1H were observed due to brain hemisphere, gender or when diagnostic groups were subdivided according to antidepressant use. Possible differences in GR mRNA expression associated with smoking were analysed using the 65 cases for whom smoking status around time of death was known. There was no evidence of a significant main effect of smoking at time-of-death on pan GR, GR-1B, GR-1C or GR-1H mRNA levels. Within schizophrenia cases only, pan GR and GR-1H mRNA levels were significantly correlated with duration of illness (r = −0.43, *p*<0.05 and r = −0.39, *p*<0.05 respectively). GR-1B and GR-1C transcripts were not correlated with duration of illness. None of the transcripts were significantly correlated with age of onset or fluphenazine-equivalent lifetime antipsychotics within schizophrenia or bipolar disorder groups.

### NR3C1 gene polymorphisms in schizophrenia and bipolar disorder

In order to explore genetic variation within the NR3C1 gene in schizophrenia and bipolar disorder, the cohort was genotyped for 11 functional or promoter NR3C1 SNPs (Table 2). Two SNPs, rs10482614 and rs6196 (N766N) showed significant genotype frequency distortion via the Hardy-Weinberg equilibrium test. The genotype frequency distortion of both these SNPs in the entire cohort was driven by distortion in the schizophrenia group only, with no significant distortion evident in control or bipolar disorder groups. SNP rs1800445 (N363S) was non-polymorphic in this largely Caucasian cohort.

**Table 2:** Details of NR3C1 SNPs genotyped in this study.

dbSNP rs #	location (UCSC build Hg19, Feb 2009)	location, description, (common name)	SNP (major/ minor allele)	MAF	HWE *p*-value
rs10052957	142786701	promoter; 2656 bases upstream of exon 1B (Tth111l)	C/T	0.320	1
rs72801094	142785905	promoter; 1860 bases upstream of exon 1B	A/G	0.046	0.177
rs5871845	142783949	5′ UTR; exon 1B	-/C	0.052	1
rs10482614	142782402	promoter; between exon 1C and exon 1H	G/A	0.134	0.012
rs10482616	142781567	promoter; between exon 1H and exon 2	G/A	0.113	0.342
rs4634384	142780697	promoter; between exon 1H and exon 2	G/A	0.490	0.687
rs6190	142780337	exon 2; non-synonymous (R23K)	G/A	0.021	1
rs1800445	142779311	exon; non-synonymous (N363S)	A/G	0	-
rs41423247	142778575	intron B; between exons 2 and 3, 647 bases downstream of exon 2 (Bcl1)	C/G	0.345	0.825
rs6196	142661490	exon 9α; synonymous (N766N)	T/C	0.139	0.017
rs6198	142657621	3′ UTR; inside exon 9β (A3669G)	A/G	0.170	0.065

Abbreviations: UCSC- University of California Santa Cruz, MAF- minor allele frequency, HWE- Hardy-Weinberg Equilibrium.

### Relationships between NR3C1 gene polymorphisms and expression of GR mRNA transcripts and protein isoforms

**Figure 5:**
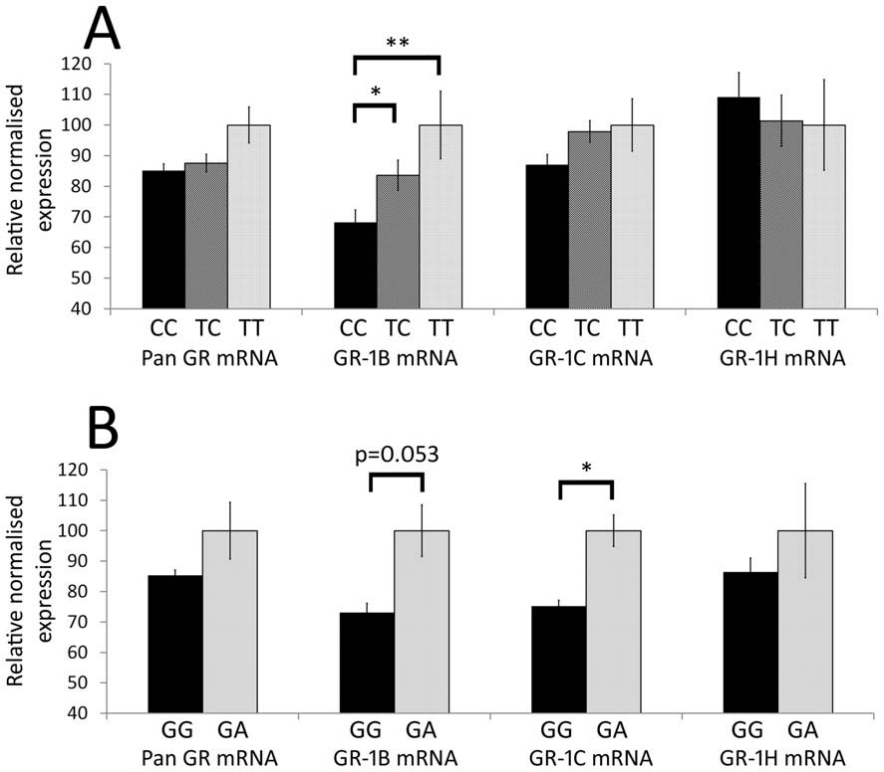
Effects of rs10052957 (Tth111l) and rs6190 (R23K) genotypes on GR mRNA expression. A) A significant main effect of rs10052957 genotype on GR-1B mRNA expression was identified (ANCOVA F(2, 81) = 5.32, *p*<0.001), with significant decreases in GR-1B expression in individuals with CC genotype (n = 46) relative to those with TC genotype (n = 40; 18.4% decrease, *p*<0.05) and TT genotype (n = 9; 31.8% decrease, *p*<0.005). B) An effect of rs6190 genotype on GR-1C mRNA expression was also observed (Mann-Whitney U-test, z = 2.58, *p*<0.01), with a 24.8% decrease in GG homozygotes (n = 91) relative to GA heterozygotes (n = 4). Error bars represent SEM. * *p*<0.05, ** *p*<0.005.

A significant main effect of rs10052957 (Tth111l) genotype on GR-1B mRNA expression was found (ANCOVA F(2, 81) = 5.32, *p*<0.001; Figure 5A). There was not a significant genotype×diagnosis interaction. By LSD post-hoc test, the GR-1B mRNA expression of individuals with CC genotypes (n = 46) was 18.4% lower than those of individuals with TC genotypes (n = 40; *p*<0.05), and 31.8% lower than those with TT genotypes (n = 9; *p*<0.005). Thus, an allele dose effect on GR-1B mRNA expression was observed. No significant main effects of rs10052957 genotype on pan GR, GR-1C or GR-1H mRNA expression were seen (all *p*>0.11).

For the rs6190 (R23K) SNP, a significant effect of genotype on expression of GR-1C mRNA in the DLPFC was also observed (Mann-Whitney U-test, z = 2.58, *p*<0.01; Figure 5B). The GR-1C mRNA expression of GG homozygotes (n = 91) was 24.8% lower than that of GA heterozygotes (n = 4). A trend towards a main effect of rs6190 genotype on expression of GR-1B mRNA was observed (Mann-Whitney U-test, z = 1.94, *p* = 0.053). There was no relationship between rs6190 genotype and expression of pan GR or GR-1H mRNA transcripts.

**Figure 6:**
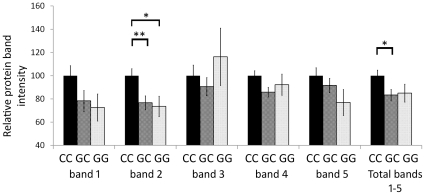
Effect of rs41423247 (Bcl1) genotype on GRα protein expression in the DLPFC. There were significant main effects of genotype on IR band 2 intensity (ANCOVA, F(2, 87) = 4.85, *p*<0.05) and total IR bands 1–5 (ANOVA F(2, 86) = 3.28, *p*<0.05). The abundance of IR band 2 (67 kDa GRα) was significantly decreased in rs41423247 GG homozygotes (n = 11; 26.5% decrease, *p*<0.05) and GC heterozygotes (n = 43; 23.4% decrease, *p*<0.01) relative to CC homozygotes (n = 41). The total GRα abundance (IR bands 1–5) was decreased (15.1%) in GC heterozygotes relative to CC homozygotes (*p*<0.05). * p<0.05, ** p = 0.006.

Levels of GRα protein isoforms in the DLPFC have been quantified and reported previously [3], and here were analysed according to NR3C1 genotype as described above for GR mRNA transcripts. In the DLPFC, five GRα immunoreactive (IR) bands are consistently observed, with IR band 1 corresponding to full-length GRα, IR band 2 to a 67 kDa GRα isoform, IR bands 3 and 4 to putative GRα-D N-terminal isoforms, and IR band 5 to a 25 kDa GRα isoform [3]. Significant differences in DLPFC GRα IR band intensity according to rs41423247 (Bcl1) genotype were identified (Figure 6). For rs41423247, significant main effects of genotype on IR band 2 intensity (ANCOVA, F(2, 87) = 4.85, *p*<0.05) and total IR bands 1–5 (ANOVA F(2, 86) = 3.28, *p*<0.05) were seen. There were no significant genotype×diagnosis interactions. By LSD post-hoc, IR band 2 (67 kDa GRα) abundance was significantly decreased in rs41423247 GG homozygotes (n = 11; 26.5% decrease, *p*<0.05) and GC heterozygotes (n = 43; 23.4% decrease, p = 0.006) relative to CC homozygotes (n = 41). A 15.1% decrease in total intensity of IR bands 1–5 in GC heterozygotes relative to CC homozygotes was also observed (*p*<0.05). No effects of rs4142324 genotype on IR bands 1, 3, 4 or 5 were seen. No additional differences in GRα IR band intensity were detected when samples were grouped according to the other genotyped SNPs.

## Discussion

In this study, we identified abnormalities of GR mRNA expression in schizophrenia and bipolar disorder, which we describe for first time in the context of variation within the NR3C1 gene. In the DLPFC in schizophrenia cases, there was decreased expression of pan GR mRNA and at least two mRNA variants, GR-1B and GR-1C, which use alternative promoters. In bipolar disorder, GR mRNA deficits were more limited, and appeared to impact the GR-1C but not GR-1B mRNA transcript. Changes in GR-1H mRNA were not conclusive, given that three schizophrenia cases potentially obscured what would have otherwise been significant decreases in GR-1H mRNA in both schizophrenia and bipolar disorder.

These findings confirm and extend prior observations of GR mRNA abnormalities in both schizophrenia and bipolar disorder. Previous exploration of GR mRNA expression using a pan GR riboprobe revealed a significant decrease in total GR mRNA in the DLPFC in schizophrenia relative to controls [2]. In that study, the decrease in total GR mRNA in bipolar disorder failed to reach significance relative to controls, being less substantial than the decrease observed in schizophrenia. The findings in this study are consistent with that previous report, with significant decreases in total GR mRNA in schizophrenia relative to both controls and bipolar disorder cases, but only a small non-significant average decrease in bipolar disorder relative to controls. By using primers/probes specific to GR exon 1 mRNA transcript variants in this study, we were able to determine that decreases in total GR mRNA in schizophrenia arise due to combined deficits of more than one GR exon 1 transcript variant. We were also able to confirm that a specific GR mRNA deficit, restricted to the GR-1C transcript, does exist in the DLPFC in bipolar disorder, although this is not reflected in significantly decreased total GR mRNA expression. This GR-1C deficit may be masked by other unchanged transcripts (such as GR-1B) when quantifying total GR mRNA in bipolar disorder. Indeed, our correlational analysis suggested that GR-1B mRNA levels accounted for ∼48% of variance in pan GR mRNA levels in the DLPFC.

Our results also suggest that functional polymorphisms within the GR (NR3C1) gene may impact GR gene expression. In particular, GR-1B mRNA transcript variant expression may be influenced by the rs10052957 (Tth111l) SNP. Carriers of the rs10052957 major C allele displayed decreased GR-1B mRNA expression in an allelic dose-dependent fashion. The rs10052957 SNP lies most proximal to the GR-1D transcription start site, but still upstream of the alternative exon 1B, and is 793 bases upstream of the putative NR3C1 proximal promoter region [21], [22]. It is possible that, located adjacent to the NR3C1 proximal promoter region, the C and T alleles of rs10052957 may differentially facilitate GR-1B transcript expression. A protective effect of the rs10052957 minor T allele has previously been reported in bipolar disorder [12], with a lower frequency of the CT genotype in bipolar disorder cases than controls, in a cohort of 245 cases and 320 controls. Seasonal patterns of hypomania in bipolar disorder have also been associated with a haplotype containing the rs10052957 SNP [23]. In our current study, allele dose-dependent regulation of GR-1B expression by rs10052957 was not specific to schizophrenia cases, but was also observed in bipolar disorder cases and controls. It is possible that rs10052957 genotype may have played a role in GR-1B mRNA down-regulation in schizophrenia, since the major C allele of the rs10052957 SNP was over-represented in schizophrenia cases in our cohort (data not shown). However, because of the small size of our cohort, it is not possible to conclude that the rs10052957 SNP is contributing to decreased GR-1B mRNA expression in schizophrenia cases relative to controls. Further work in larger genetic samples will be needed to determine the possible contribution of the rs10052957 SNP to altered GR-1B mRNA levels in schizophrenia.

A second SNP, rs6190 (R23K), was also implicated in regulation of GR mRNA expression. Individuals homozygous for the rs6190 major G allele (SNP non-carriers) displayed significantly lower GR-1C mRNA expression than individuals carrying the minor A allele (SNP carriers). The rs6190 SNP is a non-synonymous coding SNP located in codon 23 of exon 2, which results in the substitution of an arginine (R) residue for a lysine (K) in the translated receptor amino acid sequence [24]. It is often referred to as ER22/23EK because it is linked to rs6189, a synonymous SNP, in codon 22 [which codes for glutamic acid (E)]. In bipolar disorder patients, the rs6190 SNP may impact illness course, with carriers of the rs6190 SNP displaying an 8 year earlier onset of their first hypomanic episode than non-carriers [23]. Previous work has also revealed increased frequency of rs6190 carriers in major depression [13], [14], and somewhat paradoxically, a faster response to antidepressant treatment among rs6190 carriers. It is not clear how the R23K amino acid change impacts the cellular actions of the receptor, or by what mechanism it may alter the onset and course of bipolar disorder or confer vulnerability to major depression. However, since the amino acid substitution occurs in the primary receptor transactivation domain, it is possible that the transcriptional activation of target genes by the receptor may be altered by the SNP. The mechanism by which rs6190 genotype may alter GR mRNA transcript expression is not known.

We also report a main effect of the rs41423247 (Bcl1) SNP on abundance of the 67 kDa GRα protein isoform. Carriers of the minor G allele of rs41423247 displayed decreased 67 kDa GRα isoform abundance relative to CC homozygotes. The rs41423247 SNP is located in intron B (between exons 2 and 3), 645 bases downstream of exon 2. Rs41423247 has been shown to modulate HPA axis function, with G carriers reported to have higher baseline and peak ACTH levels and diminished responses to antidepressants [25]. In addition, as part of a haplotype with rs33388 and rs33389 in intron B, healthy G carriers display lower post-dexamethasone cortisol levels [26]. GG homozygotes have also been reported to have lower cortisol responses to psychosocial stress, and be at increased risk of major depression [7], [13]. Decreased abundance of the predominant DLPFC GRα receptor isoform (67 kDa GRα) in GG homozygotes and G allele carriers would be consistent with these findings, and may suggest a possible mechanism though which rs41423247 genotype may impact HPA axis function. Given that the 67 kDa GRα isoform was not altered in schizophrenia and no diagnosis×genotype interaction was observed, the rs41423247 polymorphism may be mediating effects on HPA axis activity in general, independent of the pathogenesis of schizophrenia or bipolar disorder.

Increased expression of pan GR, GR-1B and GR-1H mRNA transcripts in suicide-positive schizophrenia cases relative to suicide-negative schizophrenia cases was observed. This finding replicates earlier qPCR results with pan GR mRNA in a different schizophrenia cohort [3]. In our current study, a relationship between suicide and GR mRNA expression was prominent in schizophrenia, but limited to the GR-1C transcript variant in bipolar disorder. Previous work, employing *in situ* hybridisation in bipolar disorder cases, has also identified increased GR mRNA expression in suicide-positive relative to suicide-negative bipolar disorder cases in a third cohort [2]. Whether increased GR mRNA expression is a biological risk factor for suicide in psychotic illness, or reflects an interaction of illness pathology with altered stress experiences prior to death by suicide remains to be determined. The observed effect of suicide was most acute in the GR-1H mRNA transcript variant. The two highest GR-1H mRNA expressing schizophrenia cases, with substantially increased expression levels (Figure 3), were suicide-positive, as were four other high-expressing cases. Therefore, the differences in GR-1H mRNA between diagnostic groups, which failed to reach significance, may have been partially obscured by the high GR-1H mRNA expression of suicide-positive cases.

One of the challenges associated with the use of human post-mortem tissue is the possible confounding effect of factors such as antipsychotic exposure, antidepressant use and smoking. In this study, the lack of correlation between GR mRNA transcript levels and estimated lifetime antipsychotics doses suggests that decreased GR mRNA transcript expression in schizophrenia and bipolar disorder may not be a direct consequence of antipsychotic drug exposure. In our previous study, we also did not observe correlation of pan GR mRNA levels with estimated daily, last or lifetime antipsychotics doses [3]. Furthermore, although *in vitro* work has characterised the effects of antidepressants on GR transcription and function [27], [28], such effects of antipsychotics on GR have not been established [27]. In this study, when effects of antidepressants were explored by subdividing and analysing the schizophrenia and bipolar disorder groups according to antidepressant use, there were no significant differences between antidepressant-positive and antidepressant-negative cases. No significant main effects of smoking on GR mRNA expression were seen. Further work is required to clarify the potential effects of these confounding factors on GR mRNA expression in the prefrontal cortex.

In this study, we observed that decreased GR-1C mRNA expression was common to both schizophrenia and bipolar disorder, while decreased pan GR and GR-1B mRNA expression were present only in schizophrenia. This evidence is consistent with other molecular, epidemiological and clinical evidence, which also highlights similarities and differences between schizophrenia and bipolar disorder. Psychotic features are common to both bipolar I disorder and schizophrenia, as are some neuropathological changes such as reduced hippocampal volume [29]. Some early life experiences, such as early parental loss and childhood abuse, are common risk factors for both schizophrenia and bipolar disorder [30]. Abnormal cortisol secretion and dexamethasone suppression have been observed in both disorders [5], [6], [31]. At the molecular level, numerous mRNA transcripts and proteins are altered in both schizophrenia and bipolar disorder, including brain-derived neurotrophic factor (BDNF), glutamic acid decarboxylase-67 (GAD-67) and growth-associated protein-43 (GAP-43) [32]–[38]. Some of these transcripts, such as BDNF and GAP-43, are down-regulated by cortisol [39]–[41]. Increased abundance of the truncated GRα-D1 isoform has been shown in the DLPFC in both schizophrenia and bipolar disorder [3]. Some common genetic factors have been shown to underlie schizophrenia and bipolar disorder [42]. More work is required to understand whether common patterns of GR expression (potentially involving the GR-1C mRNA transcript) are related to common risk factors for, or symptoms of, the two disorders. Schizophrenia and bipolar disorder also display distinctive features. Affective symptoms are displayed in bipolar disorder but not necessarily schizophrenia. Some early life experiences, such as obstetric complications, are risk factors for schizophrenia but possibly not for bipolar disorder [43], [44]. A number of neuropathological changes, such as increased DLPFC neuron density, are observed in schizophrenia but have not been detected in bipolar disorder [45], [46]. Further work is also required to determine whether the divergent patterns of GR mRNA expression (potentially involving the GR-1B mRNA transcript) in schizophrenia and bipolar disorder are related to differences between the illnesses.

Overall, we provide further evidence of GR mRNA abnormalities in schizophrenia and bipolar disorder, which involve the GR-1B, 1C and 1H mRNA transcript variants. Our data also suggests the potential involvement of NR3C1 polymorphisms, rs10052957 (Tth111l), rs6190 (R23K) and rs41423247 (Bcl1), which may play a role in regulating the expression of the GR-1B mRNA transcript, GR-1C mRNA transcript and 67 kDa GRα protein isoform respectively. Experimental work in cell culture systems may clarify regulatory effects of the NR3C1 polymorphisms on GR mRNA transcripts and GRα protein isoforms. Further work in living people with schizophrenia and in larger genetic samples will also be needed to determine the link between GR gene variants, altered brain mRNA levels and changes in stress responsiveness in schizophrenia and bipolar disorder. Our data provide further information on which genetic variants are likely to be the most informative for future clinical studies.

## Materials and Methods

### Tissue collection

These studies were carried out in accordance with the declaration of Helsinki, after approval by the Human Research Ethics Committee at the University of NSW (#HREC07261). Cases from the Stanley Foundation Array cohort were used (Table 1). Genomic DNA and total RNA were extracted by the Stanley Medical Research Institute (SMRI) from tissues from the middle frontal gyrus. Genomic DNA was extracted using the Promega Wizard genomic kit (Promega, Madison, WI) using DLPFC tissues from 34 schizophrenia cases, 30 bipolar cases and 31 controls. Total mRNA was extracted with Trizol reagent (Invitrogen, Carlsbad, CA) as previously described [47], using tissues from all 35 schizophrenia cases, 31 bipolar cases and 34 controls. The quality of extracted total RNA was determined by SMRI using the Agilent Bioanalyzer 2100 (Agilent Technologies, Palo Alto, California). RNA quality was indicated by an RNA integrity number (RIN) ranging from 0 (degraded) to 10 (very good quality), and did not differ between diagnostic groups. Extracted genomic DNA and total RNA were supplied by SMRI for subsequent experiments.

### PCR detection of GR exon 1 variants

Endpoint PCR was performed to amplify GR exon 1 mRNA transcript variants in schizophrenia, bipolar disorder and control cDNA (pooled from all cohort samples) from DLPFC tissue. For targets not amplified in DLPFC cDNA, reactions were repeated with universal human cDNA from normal human tissues (Biotaq, Gaithersburg, MD). Primer sequences and reaction conditions are shown in Table 3. Each reaction contained MgCl_2_ (2–4 mM), dNTPs (0.2 mM), forward and reverse primers (0.2 mM), cDNA (approximately 4.5 ng/µl) and RedHot DNA polymerase (0.5 U; Thermo Scientific, Waltham, MA) in 1× reaction buffer. For PCR, the reaction mix including cDNA was incubated at 94°C for 3 min, followed by 40 cycles of 94°C (30 s), 53–62°C (30 s, or 90 s for GR-1Atotal) and 72°C (30 s), then 72°C for 10 min and 4°C overnight. Products were run on a 1% agarose gel alongside a 1 kb ladder (Fermentas, Waltham, MA), and visualised on the Chemidoc XRS Molecular Imager (Bio-Rad, Hercules, CA).

**Table 3:** Endpoint PCR primers used for detection of GR exon 1 mRNA transcript variants.

target transcript	reference	annealing temps	MgCl_2_ (mM)	forward primer sequence	reverse primer sequence
GR-Atotal	Breslin 2001	56–60	2	ATCACTTTCACTTCTGCTGG	CAGTGGATGCTGAACTCTTGG
GR-1B	Turner 2005	55	2	GCCGGCACGCGACTCC	CAGTGGATGCTGAACTCTTGG
GR-1Ctotal	Turner 2005	53	2	GCTCCTCTGCCAGAGTTGAT	CAGTGGATGCTGAACTCTTGG
GR-1C2	Turner 2005	55	2	TCTGTCTGTGACGGATTCTGC	CAGTGGATGCTGAACTCTTGG
GR-1C3	Turner 2005	53	2	CTTAAATAGGGGCTCTCCCC	CAGTGGATGCTGAACTCTTGG
GR-1D	Turner 2005	54–58	3	ACAACCTTTCCCAGAGTC	CAGTGGATGCTGAACTCTTGG
GR-1E	Turner 2005	53–62	2	CGTGCAACTTCCTTCGAGT	CAGTGGATGCTGAACTCTTGG
GR-1F	Turner 2005	55–60	3	GTAGCGAGAAAAGAAACTGG	CAGTGGATGCTGAACTCTTGG
GR-1H	Turner 2005	59	4	CTGACAGCCCGCAACTTGGA	CAGTGGATGCTGAACTCTTGG

### Quantitative real-time PCR (qPCR) and endpoint PCR analysis

For GR mRNA transcript quantification, qPCR analysis was conducted as previously reported [48]. Briefly, cDNA was synthesised from 3 µg total RNA from DLPFC tissue, using the Superscript First-Strand Synthesis Kit (Invitrogen). Pre-designed Taqman gene expression assays (Applied Biosystems, Foster City, CA) targeting pan GR, GR-1B and GR-1C_total_ (henceforth simply GR-1C) and GR-1D were used along with four ‘housekeeper’ genes: β-actin (ACTB), beta-2-microglobulin (B2M), TATA-binding protein (TBP) and ubiquitin C (UBC). In addition, custom Taqman primer/probes designed to target the exon 1–2 boundary of GR-1E, GR-1F and GR-1H were also used (Table 4). No Taqman primer/probe for GR-1A_total_ (henceforth simply GR-1A) was designed or tested. Serial dilutions of cDNA, pooled from all cohort samples, were included on every qPCR plate for quantification of sample expression by the relative standard curve method. ‘No template’ and ‘no reverse transcriptase’ controls were included on each plate to rule out non-specific amplification.

**Table 4:** Taqman primers/probes used for qPCR analysis of pan GR mRNA and GR exon 1 mRNA transcript variants. HK- housekeeping gene.

	Taqman accession	probe location (exon boundary)	assay function	forward primer sequence	probe sequence	reverse primer sequence
ACTB	Hs99999903_m1	1 - 1	HK	-	-	-
B2M	Hs99999907_m1	2 - 3	HK	-	-	-
TBP	Hs00427620_m1	2 - 3	HK	-	-	-
UBC	Hs00824723_m1	1 - 2	HK	-	-	-
pan GR	Hs00230818_m1	7 - 8	target gene	-	-	-
GR-1B	Hs01005211_m1	1 - 2	target gene	-	-	-
GR-1C	Hs01010775_m1	1 - 2	target gene	-	-	-
GR-1D	Hs03666144_m1	1 - 2	target gene	-	-	-
GR-1E	-	1 - 2	target gene	CGCTGGAGGTTTTGCATTTGG	CCTTCGAGTTGATATTCAC	CAGGAGTTAATGATTCTTTGGAGTCCAT
GR-1F	-	1 - 2	target gene	CCGCCGCCACCCTTT	TATCAACCCCCAACTCCC	CAGGAGTTAATGATTCTTTGGAGTCCAT
GR-1H	-	1 - 2	target gene	GCGTGTCGGAGAGAGAACT	TCCATCAGTGAATATCAACTGTT	GGGTTTTCTTCTCTACCAGGAGTTA

For qPCR gene expression analysis, each sample was assayed in triplicate. Raw expression data were normalised to the geometric mean of four housekeeper genes, and population outliers excluded if their normalized expression values were greater than 2 standard deviations from the group mean. The raw expression levels of the four housekeeper genes did not vary according to diagnosis when analysed by ANOVA [TBP- F(2, 96) = 0.30, *p* = 0.74; UBC- F(2, 96) = 0.93, *p* = 0.40; ACTB- F(2, 96) = 0.70, *p* = 0.50; B2M F(2, 96) = 0.10, *p* = 0.90]. For each analysis, between 32–34 control cases, 29–31 bipolar disorder cases and 31–34 schizophrenia cases were retained after outlier removal.

### Western blotting

Western blotting was performed using crude protein homogenates from the DLPFC of 35 schizophrenia cases, 34 bipolar cases and 35 controls. Seven micrograms of protein homogenate was heated (95°C, 5 min), run on 10% bis-tris polyacrylamide gels (Bio-Rad) and transferred onto nitrocellulose membranes (Bio-Rad) at 100 V for 30 or 120 min. Blots were probed with anti-GRα primary antibody (1∶2000 dilution in 5% skim milk, sc-1002X, Santa Cruz Biotechnology, Santa Cruz, CA), followed by goat anti-rabbit secondary (1∶2000; Millipore, Billerica, MA). After stripping (stripping buffer: 25 mM glycine, 1.5% sodium dodecyl sulfate, pH 2.0), blots were incubated with anti-β-actin primary antibody (1∶10 000; MAB1501, Millipore), followed by goat anti-mouse secondary (1∶5000; Millipore).

All blots were imaged using the Chemidoc XRS Molecular Imager (Bio-Rad). A shorter exposure time (10 s) was used to quantify the abundant 67 kDa IR band, whereas a longer exposure time (90 s) was used to quantify total GRα protein and all other IR bands. All GRα IR bands were within a linear range of detection. Duplicate samples were run in separate experimental runs. Within each run the total intensity of each immunoreactive band was normalised to an internal control (pooled sample from entire cohort) loaded onto the same gel, and to the β-actin band detected in the same lane. Population outliers in each diagnostic group were excluded if the sample normalised quantity value was greater than 2 standard deviations from the group mean. The geometric mean of both runs was then calculated, expressed as a percentage of the control mean for each band. A minimum of 33 cases per diagnostic group for each immunoreactive band were analysed. Overall, the internal standard displayed an average variability of +/−11.5% from blot-to-blot. The methods and data from western blotting experiments analysed in this study have been previously described [3].

### Statistical analysis

All data were normally distributed. For all mRNA measures, Pearson correlation analyses were conducted with possible confounding demographic factors including age, pH, PMI and RIN, and additionally within schizophrenia and bipolar disorder cases on age-of-onset, duration-of-illness and fluphenazine-equivalent antipsychotic drug measures. If significant correlations with demographic variables were observed, analysis of covariance (ANCOVA) was used with significantly correlated demographic variables as covariates, to identify effects of diagnosis, gender, manner of death, and antidepressant use. Planned comparisons of schizophrenia, bipolar disorder and control groups were made using Fisher's LSD post-hoc test. Analyses of variance (ANOVAs) were used if no correlations were seen. Two-way ANOVAs were also used, with diagnosis, genotype and smoking status as independent factors, to identify the effects of genotype and smoking on mRNA expression, and also identify any genotype-diagnosis interactions. For SNPs rs6190 and rs6196, which had genotype groups containing fewer than five cases, non-parametric tests (Mann-Whitney U-tests and Kruskal-Wallis tests) were used to identify group differences in mRNA expression according to genotype.

### Genotyping

Eleven coding, promoter or putative functional single nucleotide polymorphisms (SNPs) in the NR3C1 gene were chosen for genotyping (Table 4). Genotyping was performed at the Australian Genome Research Facility, with 20 ng of genomic DNA, in a multiplex assay using a Sequenom MassArray, Autoflex Spectrometer and iPLEX GOLD chemistry. The pass rate for genotyped samples was 99.0%. Genotyping of one SNP, rs10482605 (NR3C1-1), failed to produce any results. PLINK software (version 1.06, http://pngu.mgh.harvard.edu/purcell/plink) [49] was used for Hardy-Weinberg equilibrium testing.
